# Measuring the energy landscape: an experimental approach to the study of buckling in thin shells

**DOI:** 10.1098/rsta.2022.0027

**Published:** 2023-04-03

**Authors:** Sagy Lachmann, Shmuel M. Rubinstein

**Affiliations:** Racah Institute of Physics, Hebrew University of Jerusalem, Jerusalem, Israel

**Keywords:** buckling, energy landscape, stability landscape, ridge tracking, lateral probing, poking

## Abstract

Recent research into the buckling load of thin shells has focused on local poking of the shell. In this approach, the shell is poked under load to extract its *stability landscape*, and a *ridge tracking* method is performed to estimate the buckling load of the shell. It is the current understanding that the stability landscape measures the local stability of the shell and, as a result, that the accuracy of ridge tracking greatly relies on the location of poking. Currently, there is no method that can predict where poking should be performed on an experimental system. Here, we examine the global response of thin shells to poking through the energy landscape. We present an experimental method for measuring the energy landscape of thin shells and demonstrate its application on a thin plate strip. We show that by analysing the dynamics of the shell in the energy landscape we can experimentally measure the buckling mode of the system, which gives the correct point of poking for accurate ridge tracking, and identify two kinds of buckling points. Finally, we propose how this approach can be applied to more complex systems such as thin cylinders.

This article is part of the theme issue ‘Probing and dynamics of shock sensitive shells’.

## 1. Introduction

Estimating the buckling load of thin shell structures is a topic of great interest. Despite this, the defect sensitivity of these systems has made their study very difficult [1–9]. Historically, this difficulty was addressed by characterizing a conservative lower boundary of the knock-down factor as a design criterion [1,2]; however, this approach leaves much to be desired in terms of characterizing an individual structure in a non-destructive manner.

Recently, there has been an emerging interest in examining the fully nonlinear, out of equilibrium behaviour of thin shells using imposed numerical and physical constraints. In this context, buckling is a non-continuous change in the steady-state shape of the structure in response to a change in the applied constraints. Horák *et al.* [10] numerically explored the energy landscape of cylindrical shells—the configuration space of the system with the associated energy of each configuration. Using a ‘mountain pass’ algorithm they found the lowest energy path to buckling which goes through a *localized* equilibrium mode, unlike the global modes that are obtained from linear stability analysis [11,12]. Kreilos & Schneider [13] expanded on this result and computed the force equilibrium solutions of a thin cylindrical shell, finding localized unstable modes at the boundary of the basin of attraction of the undeformed state. Moreover, they showed that these localized modes are edge-states of the system; the basin of attraction of the system is defined as the set of all points that return to the rest state of the system and edge-states of the system are attractors of the dynamics along the boundary of the basin. Virot *et al.* [9,14] used lateral poking to study the stability of thin cylindrical shells under finite amplitude perturbations. By poking the cylinder at different axial loads they were able to identify a ‘stability landscape’ which captures the stability of a system, with the transition from a positive force on the poker to a negative force at buckling. It was then shown by Abramian *et al.* [15] that by performing a ‘ridge tracking’ procedure on these landscapes, one can estimate the buckling load of a cylindrical shell with an introduced defect to within 5% error. Hutchinson & Thompson [16] suggested that the work done by the poker is in fact the energy barrier to buckling through lateral deformation; a zero ridge height coincides with a zero energy barrier to buckling, thus, the ridge tracking method estimates the axial load where spontaneous buckling is initiated. The stability landscape has recently been used by Royer & Pellegrino [17] to look at optimal design for space-deployable structures and study the early transition into the post-buckled regime. A similar approach was also recently explored by Marthelot *et al.* [18] and Abbasi *et al.* [19] to accurately predict the buckling of hemispherical shells. It was reported that proper selection of the point of probing on the shell is paramount for an accurate prediction. It is thus the current understanding that ridge tracking measures the stability of the system to finite amplitude perturbations at a specific location, however, knowing the right location to poke is extremely challenging, as highlighted by Ankalhope & Jose [20]. This would suggest that an approach that looks at more than just the local response could be beneficial for the study of the stability of thin shells.

The configuration of a buckling system as a whole has been mostly limited to theoretical exploration with experiments focusing mainly on the local perturbation and response of the system. Neville *et al.* [21] used rigidly connected ‘pokers’ to control the shape of a shallow arch and find its unstable equilibrium points, in a manner similar to the numerical continuation method [22]. Using one poker, they move the midpoint of the system to a desired deflection, and two additional pokers are then moved to find configurations of zero force, thus experimentally tracking the edge of the basin of attraction of the system. This work nicely identifies trajectories on the basin boundary, yet it does not explore the dynamics up to the boundary or the transition into buckling. Nevertheless, the results highlight the strength of poking and shape control of thin structures in measuring their unstable behaviour.

In this paper, we take a similar approach and leverage the Hamiltonian nature of elastic thin shell structures in describing their dynamics and stability through the energy landscape. We use pokers as shape control parameters to explore the configuration space of the system and map its energy landscape. We show how the energy landscape can be measured experimentally, and how it can be used to find the buckling load and initiation point in an experimental system. As a proof of principle, the approach is demonstrated for a thin plate strip, and we describe how this method can be implemented in more defect-sensitive structures.

## 2. Experimental approach

A state space describes all the possible configurations of a given structure, from the unperturbed state to any post-buckled states and all the states between them. Focusing on elastic systems without plastic deformations, each configuration has a corresponding energy. The energy landscape is the hyper-dimensional manifold of the energy as a function of configuration. For a Hamiltonian system, in which the forces are conservative, the energy landscape determines the quasi-static dynamics of the system through its topography. In this approach, a system is stable if the steady state configuration is continuous under perturbations of the applied constraints, and it buckles when a perturbation of the constraints causes a non-continuous change in the steady-state configuration.

Assuming some decomposition of the configuration space exists, every shape can be described by a vector of coefficients w with a corresponding energy function U(w). For any small poke, dy, the system’s shape and energy change by small amounts dw and dU, respectively. In this case, the force applied on the poker F is

2.1 $$F=\frac{dU}{dy}=\nabla_{w}U\cdot \frac{\partial w}{\partial y}.$$

Thus, the force the system applies on a poker equals the local gradient of the energy landscape multiplied by the tangent to the trajectory of the system in configuration space. When the angle between the gradient and the trajectory is greater than $90^{\circ }$ the poker experiences a negative force where instead of pushing against the poker, the system is pulling on it. If the poker is not rigidly attached, the system is ‘pulled away’ as it buckles to a lower energy state, analogous to the ‘unstable lake’ region in the stability landscape [9]. A transition to buckling will, therefore, pass through a point of zero force on the poker. Note that not all configurations with a zero poker force are at a buckling point, e.g. the original undeformed state is also characterized by zero poker force.

Equation (2.1) also indicates how the relative energy of a configuration can be measured: after integrating with respect to dy, the left-hand side yields the work done by the poker while the right-hand side yields the energy of the system up to a constant. This approach generalizes to a system with multiple pokers where each poker performs some work and the energy of the entire system is the sum of all the work done by the different pokers.

Poking applies a constraint that limits the configuration space that the system can explore, forcing the system to take the lowest energy configuration that is accessible to it and is not separated by an energy barrier. In this paper, we measure the energy landscape of a shallow arch in the form of a thin plate strip using lateral pokers as *shape control parameters*. The thin plate strips we use are metallic rulers (W×L×T=26×310×1 mm) held at a constant end-to-end shortening and a simply supported boundary condition (zero moment) along the two small faces (in the W×T plane). No twisting is applied to the rulers and no constraints are applied to the faces in the L×T plane. With these boundary conditions, the plate bends only around the W-direction.

**Figure 1.**
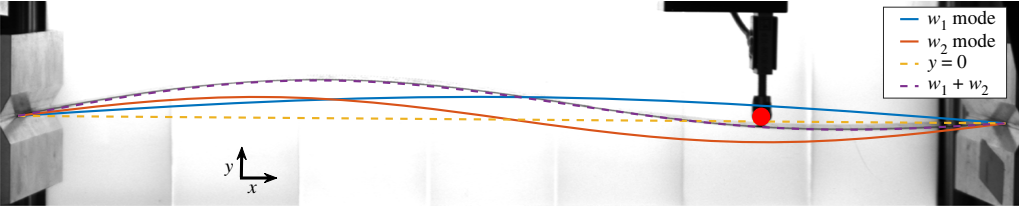
A picture of the experimental system. At the left and right there are two wedges that apply a simply supported boundary condition, between which a dashed yellow line shows the zero displacement line (y=0 mm). The red circle is the location of the poker that is in contact with the thin plate. The blue and orange lines are the decomposed first and second sine modes of the measured shape and the dashed purple line is the approximated fit of the plate’s shape, which can be seen to be a good fit of the ruler that can be seen faintly behind it. The axes of this picture are indicated by arrows with the x-axis parallel to the zero-displacement line. Note that although only one poker is shown in this figure, the system has two pokers. (Online version in colour.)

The experimental setup is based on the body of an *Instron* 1011 tensile stress tester with a DAQ based (NI USB6009), computer-controlled system. The system has two displacement-controlled metallic wedges to apply the boundary conditions. In all tests, the end-to-end displacement is kept constant at a value in which the plate exhibits a bifurcation. A combination of travel stages (*Thorlabs* LTS and *Thorlabs* MTS50) is used to move two lateral pokers in the plane perpendicular to the plate independently of one another (the x×y plane in figure 1). The pokers are round metallic spheres attached to a force gauge (*Futek* LSB200 and LSB205). The ruler is illuminated with an LED backlight to increase the contrast and its shape is visualized from the side by a camera (*LUCID* ATX081S-cc), as shown in figure 1. The centreline of the ruler is numerically extracted from these pictures and fit using a sine decomposition:

$$y(x)=\sum_{n=1}^{\infty }w_{n}\;\sin\left(\frac{n\pi x}{L}\right),$$

with L is the distance between the edges and $w_{n}$ is the decomposition coefficient of the nth mode. The zero-displacement line y=0 is chosen to be along the line between the two constrained edges of the ruler, as shown by the yellow dashed line in figure 1.

During an experiment run, the pokers are first displaced to their desired *x* position while out of contact, after which they advance toward the plate in the *y*-direction. Any probing protocol results in the system following a unique trajectory in the energy landscape. Loading protocols can be simple, with one or both pokers moving at a constant speed, or more complicated, e.g. poker 1 moves to $y_{1}$ and only after it stops does poker 2 move to $y_{2}$. As the system is Hamiltonian, its measured energy depends on its current state and not on the trajectory taken. However, trajectories may reach an unstable point before reaching their destination. Thus, to avoid buckling, the protocol is halted when the force on any one of the pokers approaches zero. A point in configuration space where buckling is initiated is called a *buckling point*.

## 3. Results

### (a) Measuring the energy landscape

**Figure 2.**
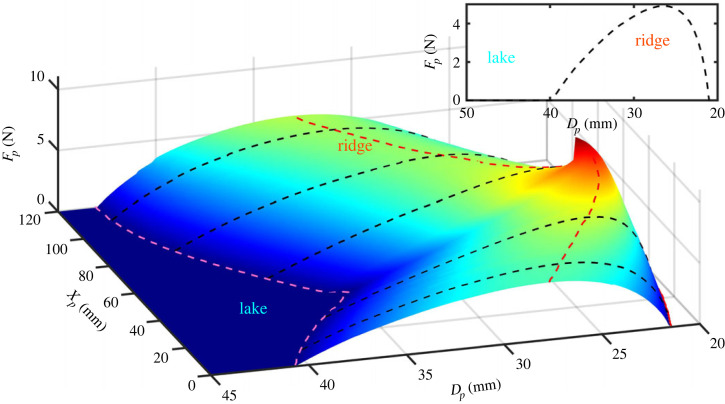
A stability landscape of a ruler. The landscape describes the measured force response of the plate ($F_{p}$), with constant end-to-end displacement boundary conditions, to poking at multiple locations. The poker displacement ($D_{p}$) and location ($X_{p}$) are the coordinate along the *x* and *y*-directions in figure 1, respectively, though $D_{p}=0$ does not correspond to y=0 and $X_{p}=0$ does not correspond to x=0. The unstable lake is identified as the region of zero force where the plate buckles and its boundary is the dashed pink line. The ridge is defined as the line of maximal force as a function of $X_{p}$ and is shown by the dashed light blue curve. The inset shows a typical example of the force response of the system at a single $X_{p}$ value along the dashed lines. (Online version in colour.)

When a single lateral poker is advanced, the force response of the system initially increases until it reaches a maximum and then decreases, as shown for a typical measurement in the inset of figure 2. To avoid buckling, as the force on the poker approaches zero, the poking is stopped. For rulers, similar force–displacement behaviour is observed at all poking locations, nevertheless, the measured values may vary significantly. Similar to the procedure suggested in [9] all the force–displacement curves are combined to outline a stability landscape, as shown in figure 2. In contrast to the stability landscape previously introduced for cylinders, which characterizes the stability of the system to poking at a given location, under changing axial loads, here the landscape describes the force response of poking at all locations under a given end-to-end displacement. Therefore, this landscape exhibits two distinct ridges and an unstable lake indicating where buckling occurs. The boundary of the unstable lake corresponds to the displacement at which buckling occurs.

**Figure 3.**
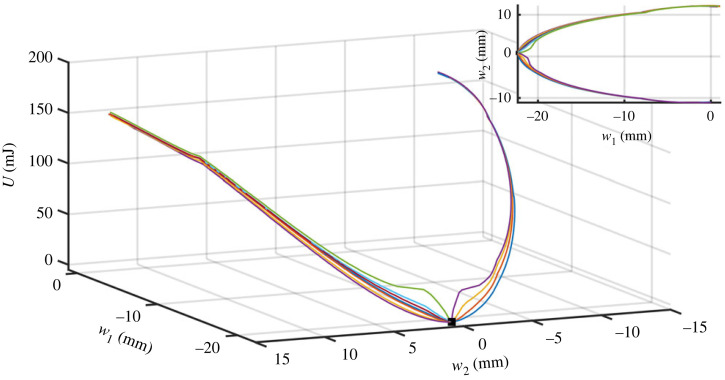
A single lateral poker at position x advances in the y-direction in contact with the plate. While the poker is displaced, its work is measured and the shape of the plate’s centreline is imaged and fit using a sine decomposition. For each x position, a $U(w_{1},w_{2})$ curve is generated which describes a trajectory in the energy landscape as a function of the first two sine coefficients of the centreline (for more details, see electronic supplementary material). The different trajectories all start at the same rest configuration (black square) and end near a buckling point. Note that by using a single lateral poker only two trajectories in the energy landscape are effectively accessible. Inset shows a projection of the same trajectories onto the horizontal plane. (Online version in colour.)

While specific force response curves are unique to the poking location, the resulting deformations are practically identical. In fact, when examining the experimental data in configuration space only two curving trajectories are observed, corresponding to poking at either side of the centre of the thin plate, as shown in the inset of figure 3. These trajectories are the collection of measured points that describe the system’s configuration and energy throughout the poking procedure, ending prior to a buckle.

Due to the simplicity of the system, its configuration space can be reduced to the first two sine components while completely describing the state of the system, see electronic supplementary material for a more detailed explanation. As the system is Hamiltonian, we would expect that the energy of the endpoints of these curves would be independent of the protocols, which is indeed seen in figure 3. These curves are trajectories in the energy landscape that result from the different protocols. This plot shows that the lake boundary is reduced to two buckling points in the energy landscape. These two buckling points are saddle points of the topography, with the trajectories leading to the *saddle trajectories*, and so it is expected that a local maximum point exists between them, though one cannot be resolved with lateral poking from a single poker.

**Figure 4.**
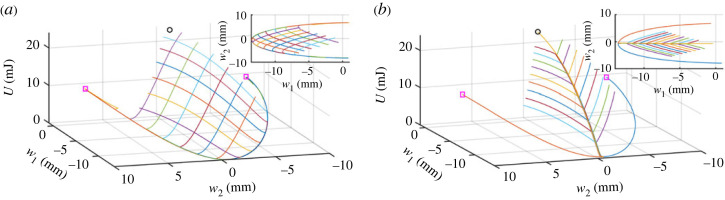
The measured trajectories in the energy landscape for two different poker protocols, highlighting the saddle points of the landscape (magenta squares) and local maximum point (black circles). In the protocols used to generate (*a*) the first poker was moved to a set location and once it reached it the second was moved in the *y*-axis until a zero force was approached. In the protocols used to generate (*b*) the first poker was moved to the same displacement and the second was moved until buckling but they both started movement at the same time. The insets are the projections of the trajectories onto the configuration space $\left(w_{1},w_{2}\right)$. It is apparent that while the two protocols measure the same energy landscape with the same energy values, the trajectories themselves and pattern on the landscape are different between the two protocols. The ‘web’ pattern is seen to better capture the vicinity of the saddle trajectories while the ‘tree’ pattern is better able to approach the local maximum. (Online version in colour.)

The ability to impose a specific shape on a system is limited by the number of control parameters and the constraints that they can apply. Using a single lateral poker, only two trajectories in the energy landscape can be measured, as shown in figure 3. When a second poker is added, a larger portion of the energy landscape is accessible. Two pokers offer far greater shape control, as shown in figure 4. Moreover, the local maximum of the energy landscape in the vicinity of $w_{1}=w_{2}=0\;\mathrm{mm}$, as well as the ridge leading up to it, are clearly resolved from the experimental data when using two pokers. In the first protocol we tested, the two pokers do not move together. The first poker is advanced to the desired displacement, only then does the second poker move in the y-direction, and is stopped when the force approaches zero. This protocol yields a ‘web’ pattern, as shown in figure 4*a*. A different poking protocol, in which both pokers are moved in the y-direction simultaneously until one is stopped at a desired y value and yields a different ‘tree’ pattern, as shown in figure 4*b*. Different poking protocols can better resolve different regions of the energy landscape, as seen by the ‘web’ pattern’s detailed mapping of the vicinity of the saddle trajectories.

### (b) Buckling initiation

In the energy landscape, any stable point as well as the buckling points can be reached by multiple, different trajectories. Nevertheless, the direction in which a buckle is initiated, $\hat{\dot{w}}$, is a unique property of each buckling point. It is common to associate the direction in configuration space in which stability is lost with the buckling mode resulting from a linear eigenmode analysis. Note that (i) the configuration space parametrization w is not necessarily the eigenmode basis at the buckling point and (ii) while there could be several unstable modes of the system for which the eigenvalue is negative, the buckling mode is the least stable mode with the lowest eigenvalue. Here, we consider a dynamic definition of the buckling mode as the experimentally measured vector in configuration space in which the buckling is initiated.

The trajectories shown in figures 3 and 4 are the set of all configurations and corresponding energies that the system exhibits throughout each poking procedure. Though it is hard to show in these plots, the rate at which the system progresses along each trajectory, d(w(t),U(t))/dt, is not constant. For a system with multiple pokers at displacements $y_{i}$, using equation (2.1) the rate of change of the configuration as a function of the energy, dw/dU, along the trajectory is

3.1 $$\frac{dw}{dU}=\sum_{i}\frac{\partial w}{\partial y_{i}}\frac{dy_{i}}{dU}=\sum_{i}\frac{dw}{dy_{i}}F_{i}^{-1}.$$

At a buckling point, at least one of the pokers will experience a zero force, $F_{j}=0$, and the rate will diverge, $dw/dU∼F_{j}^{-1}\to \infty$. A buckling point is, therefore, characterized by the rate of change of the configuration diverging along the trajectory. The zero force at buckling described by equation (2.1) is satisfied in one of two ways: (i) in the first kind of induced buckling, the system’s trajectory in the energy landscape passes through an unstable equilibrium where the gradient equals zero and (ii) in the second kind, the projection of the trajectory on the gradient changes from positive to negative, passing through a contour line where the two vectors are perpendicular. As a result, depending on the kind of buckling point, the buckling mode will either be in the direction opposite the gradient in the case of the first kind, or in the direction of a contour line in the second kind. Note that for higher dimensions of the configuration space the equivalent of the contour line will be the manifold perpendicular to the gradient at the buckling point, meaning that while there are a variety of directions in which the system could buckle, the sub-manifold that spans these directions is uniquely determined by the energy landscape at the buckling point.

The buckling points in the ‘tree’ pattern reside either at one of the saddle points or at the end of one of the ‘branches’, as seen in figure 4. These two groups of buckling points are distinct in that the saddle points are buckling points of the first kind with a buckling mode in the direction opposite the gradient, while the branch buckles are points of the second kind with a buckling mode parallel to the contour line. From the landscape measurements the buckling mode at the saddles is expected to be mainly in the $w_{1}$-direction while at the branches it will have significant components in both directions.

**Figure 5.**
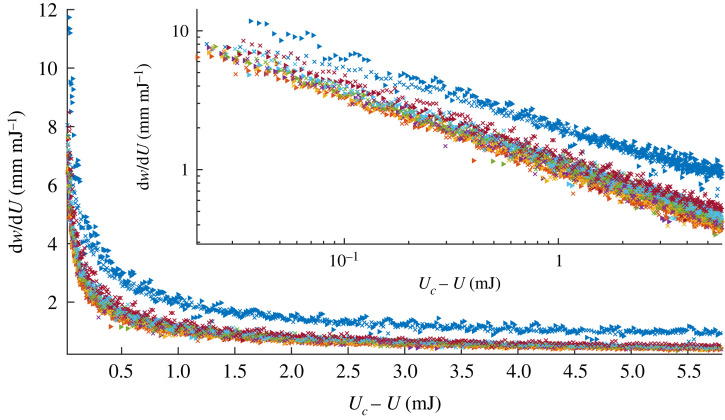
dw/dU as a function of $U_{c}-U$, where $U_{c}$ is the energy at buckling, for all trajectories shown in figure 4*a*. The blue markers correspond to the saddle trajectories. Trajectories initiating with a positive and negative $w_{2}$ component are indicated with the triangles and crosses, respectively. The blue markers are of the saddle point trajectories and the rest are branches. The inset shows the same plot in logarithmic scale. (Online version in colour.)

**Figure 6.**
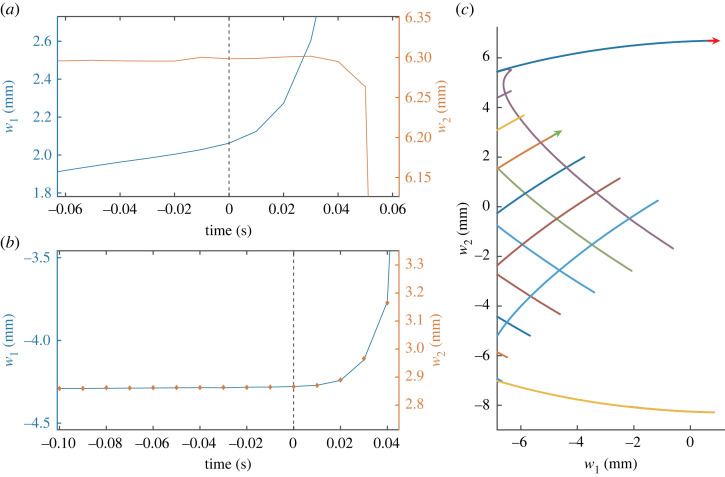
$w_{1}$ and $w_{2}$ as a function of time for two buckling points at the onset of buckling (t=0). (*a*) A branch buckle: in the first 40 ms after buckle initiation the slope of the $w_{2}$ component is negligible while the slope of the $w_{1}$ component increases significantly. (*b*) A branch buckle: the two components have a relatively constant slope ratio prior to and after buckling.(*c*) a close-up of the web trajectories in figure 4*a*. The red and green arrows show the buckling point and mode of the saddle in sub-figure (*a*) and branch in sub-figure (*b*), respectively. The arrows start at the end of the trajectory, which is the buckling point, and their direction is the tangent to the trajectory at that point, which is the buckling mode. (Online version in colour.)

Although there are two kinds of buckling points, the rate of change is as the shape diverges for both, as predicted by equation (3.1) and shown experimentally in figure 5. As for the buckling mode, examining the shape of the thin plate at the onset of buckling, $w_{1}(t)$ and $w_{2}(t)$, reveals that at the saddle point, the initial slope of the $w_{2}$ component, $\dot{w_{2}}$, is negligible and thus the buckling mode is indeed in the $w_{1}$-direction, as predicted from the energy landscape and shown in figure 6*a*. At the branch, the relative rate of the $w_{1}$ and $w_{2}$ components remains approximately constant prior to and after buckling, as shown in figure 6*b*. As the pre-buckled trajectory increases the system energy and the post buckle decreases it, it follows that the buckling mode is indeed at the boundary between the two trajectories, and is in the direction of the contourline.

## 4. Discussion and conclusion

Using a thin, metallic plate strip we demonstrate that multiple lateral pokers are effective shape control parameters for the measurement of the energy landscape, which can be used to characterize the stability of thin shell structures. With more pokers, a larger portion of the energy landscape is accessible and additional features of the topography can be discerned. Previous works pointed to the importance of the shape control parameters for evaluating the dynamic properties of a system. Neville *et al.* [21] used multiple shape controllers to sample trajectories on the boundary of the basin of attraction of the undeformed state of the system, directly relating the limitations in their measurement capabilities to the low number of independent shape control parameters. Royer & Pellegrino [17] similarly used multiple pokers with correlated motion that functioned as one non-local shape control parameter; thus, they were able to probe multiple buckling points. It may be beneficial to use the energy landscape to characterize these buckling points, as our result indicates that if all pokers experience zero force at buckling the measured buckling point is an unstable equilibrium and if only some of them experience zero force, then the system buckles through a contour line. The ability to measure unstable equilibrium points is directly tied to the number of shape control parameters used, i.e. probing an unstable equilibrium point with N unstable modes would also require N independent shape control parameters. Edge-states, which are modes embedded at the basin boundary that are unstable only in the direction of buckling [13], are thus ideal candidates for probing as they require only a single shape control parameter to identify. For example, in the simple case of the narrow plate thin plate strip explored in this work, the landscape is two-dimensional with two saddle points, one local maximum and one local minimum, with one, two and zero unstable modes, respectively. Indeed, the local minimum at the rest configuration requires no poking to be measured and we were able to measure the saddle points with a single poker while the local maximum required two pokers to be measured.

Within the energy landscape framework, there are two types of induced buckling points. The first kind is an unstable equilibrium and the second kind is a point where the trajectory becomes perpendicular to the gradient. The corresponding buckling modes are opposite to the gradient or along the contour line, respectively. Regardless of the type of buckling point, the rate of change of the configuration along the trajectory, dw/dU, diverges as the buckling point is approached. The two kinds of buckling are also distinct in their position relative to the basin of attraction, with the first kind being at the basin boundary and the second kind generally being inside the basin. The symmetries of the buckling points of the first kind, or lack thereof, are determined by the symmetry of the equilibrium points in the energy landscape, while the buckling points of the second kind generally have no symmetry. This classification of two types of buckling points is not limited to particular geometries and should be generally applicable to elastic, and Hamiltonian systems, including complex and defect-sensitive ones, e.g. a thin cylinder under axial compression. This leads to two interesting conclusions: (i) sophisticated poking protocols can be used to measure the energy landscape of real shells and (ii) probing is not strictly necessary to identify the buckling load and even the buckling mode of a system. For example, by measuring the shape of a cylinder under axial compression the buckling mode will arise as the spatial pattern whose growth rate diverges, and the buckling load will be the load for which the divergence occurs. Using the buckling mode, supplemental ridge tracking can be performed at the region of buckling initiation to validate the buckling load estimation.

A related experimental method that generated significant interest recently is the vibration correlation technique (VCT). The VCT is based on the observation that the equations for the natural frequencies of thin structures show a relationship between the natural frequency and the applied load [23]. For many beams and plates, the natural frequency approaches zero as the buckling load draws near; however, the relationship between the load and the frequency is not always known, e.g. a thin plate under shear loading has no closed form solution [24,25]. For unstiffened cylindrical shells, it was found empirically that the curve of ${\left(1-p\right)}^{2}$ as a function of $\left(1-f^{2}\right)$ is a second order polynomial, with p and f the normalized load and frequency, respectively [26]. The buckling load of the cylinder is at the local minimum point of the curve. For a VCT estimation, the frequency response of the shell is usually measured with a scanning laser vibrometer, which samples multiple points on a predetermined grid [27]. Knowing the energy landscape around the desired configuration allows us to calculate the vibration modes and frequency of the system by generating a local force equation and adding the kinetic component. Comparing this method to ridge tracking and the energy landscape method presented in this paper, ridge tracking is simpler and quicker to perform when the buckling mode is local and the initiation point is known, VCT is advantageous when the buckling load is non-local and the relationship between the load and frequency is known, and when there is no knowledge about the buckling mode the energy landscape method is best suited as it is a more general approach.

The basin of attraction of an unperturbed state is defined as all the configurations that would return to it [13]. Dynamically, assuming over-damped conditions, any initial point inside the basin would settle to the unperturbed state. This is a property of the system and its defects, regardless of the way in which it is loaded. While it would be reasonable to assume that all the points inside the basin would be stable, it is not the case. As discussed above, induced buckling of the second kind happens when the trajectory along the energy landscape is perpendicular to its gradient. This kind of buckling is a property of both the system and the specific way in which it is poked; many points within the basin of attraction can be either stable or unstable, depending on the specific constraints applied. For the thin plate strip described above, the basin boundary is given approximately by a straight line connecting the two saddle points. Almost all the buckling points measured by two poker protocols were far from this line with only the saddle buckling points being at the basin boundary, as shown in figure 4. As such, for the purpose of predicting and evaluating the system’s stability, it is not enough to know the energy landscape of the system, and by extension its basin of attraction, all the constraints on the system must be taken into account.

Recent investigations of cylindrical shells make a distinction between two types of poker-induced buckling [9]. When the lateral poking force passes the ‘ridge’ and begins to decrease, buckling is induced either when the lateral force has reduced to zero, or at some finite force. The existence of the latter makes ridge tracking more complicated as it destroys the sample without any warning. Our framework offers an appealing interpretation of these results where the types of poker induced buckling correspond to the two distinct kinds of buckling points we discussed: the zero poking force buckling is at a buckling point of the first kind at the basin boundary [13,15]. Buckling induced at a finite poking force happens within the basin of attraction at a buckling point of the second kind. Moreover, by measuring the energy landscape of the shell and tracking its trajectory, this second kind of buckling can be detected and buckling can be avoided. It is worth noting that when considering the energy barrier to buckling [16], the lowest energy barrier will be found at a buckling point of the first kind. As buckling points of the first kind are a finite set of points, there are also a finite set of energy barriers for these points. On the other hand, the energy barrier of buckling points of the second kind is determined by the exact trajectory being measured. They can take any value in the range between the lowest and highest barriers for the first kind and cannot be estimated through the stability landscape.

## Data Availability

This article has no additional data. The data are provided in electronic supplementary material [28].

## References

[1] Seide P, Weingarten VI, Peterson J. 1960 The development of design criteria for elastic stability of thin shell structures. Los Angeles, CA: TRW Space Technology Labs.

[2] Weingarten VI, Seide P, Peterson J. 1968 SP-8007.Hampton, VA: NASA.

[3] Horton W, Durham S. 1965 Imperfections, a main contributor to scatter in experimental values of buckling load. Int. J. Solids Struct. **1**, 59-62. (10.1016/0020-7683(65)90015-6)

[4] Koiter WT. 1945 The Stability of Elastic Equilibrium. Delft, The Netherlands: Delft University of Technology.

[5] Bushnell D. 1981 Buckling of shells-pitfall for designers. AIAA J. **19**, 1183-1226. (10.2514/3.60058)

[6] Hilburger Jr MW, Waters WA, Haynie WT. 2015 Buckling test results from the 8-foot-diameter orthogrid-stiffened cylinder test article ta01. Technical Report 20150017037, NASA Langley Research Center, aug.

[7] Singer J, Arbocz J, Weller T. 2002 Buckling experiments: experimental methods in buckling of thin-walled structures. Shells, built-up structures, composites and additional topics, vol. 2. Hoboken, NJ: John Wiley & Sons, Inc.

[8] Elishakoff I. 2014 Resolution of the twentieth century conundrum in elastic stability. Hackensack, NJ: World Scientific.

[9] Virot E, Kreilos T, Schneider TM, Rubinstein SM. 2017 Stability landscape of shell buckling. Phys. Rev. Lett. **119**, 224101. (10.1103/PhysRevLett.119.224101) 29286808

[10] Horák J, Lord GJ, Peletier MA. 2006 Cylinder buckling: the mountain pass as an organizing center. SIAM J. Appl. Math. **66**, 1793-1824. (10.1137/050635778)

[11] Timoshenko SP, Gere JM. 1961 Theory of elastic stability. New York, NY: Dover Publications.

[12] Brush D, Almroth B. 1975 Buckling of bars, plates, and shells. New York, NY: McGraw-Hill Book Company.

[13] Kreilos T, Schneider TM. 2017 Fully localized post-buckling states of cylindrical shells under axial compression. Proc. R. Soc. A **473**, 20170177. (10.1098/rspa.2017.0177) 28989305 PMC5627372

[14] Gerasimidis S, Virot E, Hutchinson JW, Rubinstein SM. 2018 On establishing buckling knockdowns for imperfection-sensitive shell structures. J. Appl. Mech. **85**, 091010. (10.1115/1.4040455)

[15] Abramian A, Virot E, Lozano E, Rubinstein SM, Schneider TM. 2020 Nondestructive prediction of the buckling load of imperfect shells. Phys. Rev. Lett. **125**, 225504. (10.1103/PhysRevLett.125.225504) 33315464

[16] Hutchinson JW, Thompson JM. 2018 Imperfections and energy barriers in shell buckling. Int. J. Solids Struct. **148–149**, 157-168. (10.1016/j.ijsolstr.2018.01.030)

[17] Royer F, Pellegrino S. 2022 Probing the stability of ladder-type coilable space structures. AIAA J. **60**, 2000-2012. (10.2514/1.J060820)

[18] Marthelot J, López Jiménez F, Lee A, Hutchinson JW, Reis PM. 2017 Buckling of a pressurized hemispherical shell subjected to a probing force. J. Appl. Mech. **84**, 121005. (10.1115/1.4038063)

[19] Abbasi A, Yan D, Reis PM. 2021 Probing the buckling of pressurized spherical shells. J. Mech. Phys. Solids **155**, 104545. (10.1016/j.jmps.2021.104545)

[20] Ankalhope S, Jose S. 2022 Non-destructive prediction of buckling load of axially compressed cylindrical shells using least resistance path to probing. Thin-Walled Struct. **170**, 108497. (10.1016/j.tws.2021.108497)

[21] Neville RM, Groh RMJ, Pirrera A, Schenk M. 2018 Shape control for experimental continuation. Phys. Rev. Lett. **120**, 254101. (10.1103/PhysRevLett.120.254101) 29979051

[22] Riks E. 1979 An incremental approach to the solution of snapping and buckling problems. Int. J. Solids Struct. **15**, 529-551. (10.1016/0020-7683(79)90081-7)

[23] Lurie H. 1950 *Lateral vibrations as related to structural stability*. PhD thesis, Institute of Technology, Pasadena, California, USA.

[24] Kennedy D, Lo KI. 2018 Critical buckling predictions for plates and stiffened panels from natural frequency measurements. J. Phys: Conf. Ser. **1106**, 012018. (10.1088/1742-6596/1106/1/012018)

[25] Abramovich H. 2021 The vibration correlation technique – a reliable nondestructive method to predict buckling loads of thin walled structures. Thin-Walled Struct. **159**, 107308. (10.1016/j.tws.2020.107308)

[26] Arbelo MA, de Almeida SFM, Donadon MV, Rett SR, Degenhardt R, Castro SGP, Kalnins K, Ozoliš O. 2014 Vibration correlation technique for the estimation of real boundary conditions and buckling load of unstiffened plates and cylindrical shells. Thin-Walled Struct. **79**, 119-128. (10.1016/j.tws.2014.02.006)

[27] Labans E, Abramovich H, Bisagni C. 2019 An experimental vibration-buckling investigation on classical and variable angle tow composite shells under axial compression. J. Sound Vib. **449**, 315-329. (10.1016/j.jsv.2019.02.034)

[28] Lachmann S, Rubinstein SM. 2023 Measuring the energy landscape: an experimental approach to the study of buckling in thin shells. Figshare. (10.6084/m9.figshare.c.6373227) PMC9922550 36774957

